# Spontaneous 180-Degree Rotatory Dislocation of Rotating-Platform Total Knee Arthroplasty

**DOI:** 10.1155/2019/2038983

**Published:** 2019-09-15

**Authors:** Stephen Sierra, Chibuzo Akalonu, Kevin West, Matthew Blue, George Brindley

**Affiliations:** Department of Orthopaedic Surgery and Rehabilitation, Texas Tech University Health Sciences Center, 3601 4th St. MS 9436, Lubbock, TX 79436, USA

## Abstract

Rotating-bearing total knee arthroplasty has been theorized to have some advantages in the kinematics and wear characteristics of total knee arthroplasty. A rare complication of rotating-bearing total knee arthroplasty is rotary dislocation, spinout, of the polyethylene component. When these dislocations occur, they typically result in a 90-degree dislocation in respect to the axial axis of the knee. This case is unique in that it presents a complete 180-degree polyethylene dislocation without trauma.

## 1. Introduction

Rotating-platform knee arthroplasties became popular due to their theoretical advantages relating to wear and range of motion [1]. However, one possible adverse event associated with the use of a rotating platform is subluxation or dislocation of the polyethylene. Subluxation of the articular polyethylene component of rotating-platform total knee arthroplasties (TKAs) are an uncommon, but a well-documented, complication [2]. Upon literature review, it was determined that the majority of polyethylene dislocations result in an axial 90-degree subluxation. Two reports did describe a 90-degree subluxation that underwent a full 180-degree rotation upon attempt at closed reduction [3, 4]. However, this case presents a patient that underwent spontaneous 180-degree dislocation of the polyethylene component. After thorough literature review, this appears to be the first case of a spontaneous 180-degree rotation of a rotating-platform total knee arthroplasty.

## 2. Case

A 67-y/o female (BMI: 51.19) with history of bilateral knee pain, diagnosed with osteoarthritis refractory to medical management, underwent a left total knee arthroplasty using a DePuy Synthes ATTUNE primary porous rotating-platform cruciate-retaining TKA system. Preoperatively, the patient has no instability and standing plain radiography showed a varus deformity with severe tricompartmental arthritis. The distal femur was prepared to accept a size 6 porous femoral component, and the proximal tibia was prepared to accept a size 5 cementless POROCOAT rotating-platform tibial base plate. A 5 mm size 6 tibial polyethylene articular component was placed, and the patella was prepared to accept a 35 mm all-polyethylene anatomic patellar implant. There was good ligament balance in flexion and extension as well as anatomic patellar tracking. Flexion and extension gaps were symmetric. She was discharged without complication.

**Figure 1:**
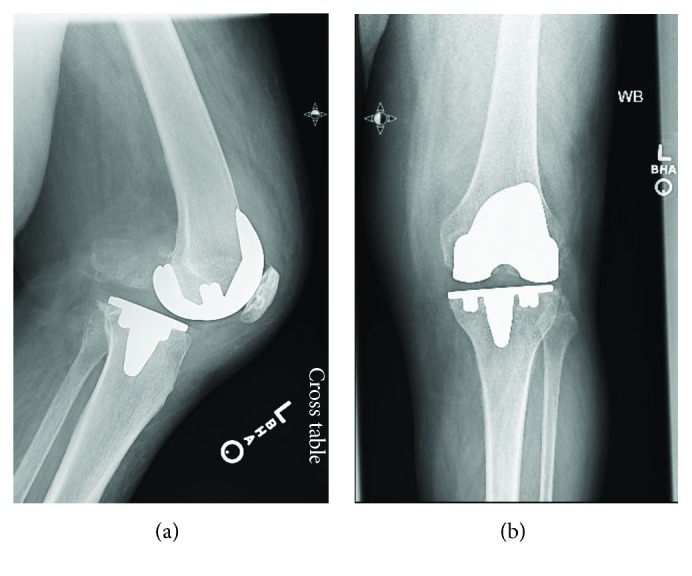
(a, b) AP and lateral views of the knee 6 weeks post-op. Normal knee architecture in both views.

**Figure 2:**
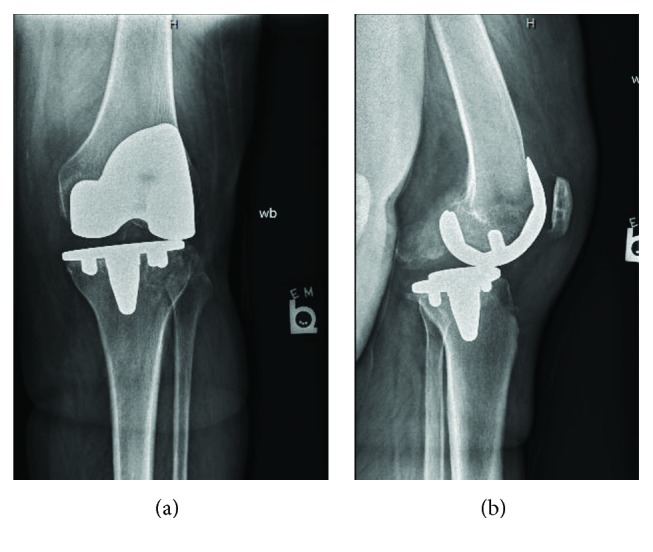
(a, b) AP and lateral radiographs of the knee indicating posterior dislocation of the tibia and instability of knee prosthesis. Note inability to ascertain the direction of the rotating platform.

Radiographs at 6 weeks post-op showed well-aligned TKA (Figures 1(a) and 1(b)). Left knee range of motion was 0–100 degrees, with no flexion/extension mismatch and normal patellar tracking. The patient did report that she had more pain from the current TKA compared to a previous right TKA. Five months postoperatively, the patient reported progressive ambulatory pain. Knee range of motion had decreased to 10-60 degrees. Radiographs showed anterior dislocation of the femoral implant on the tibial polyethylene component (Figures 2(a) and 2(b)). Open reduction verse revision of all components was planned at this time. Intraoperatively, there were no signs of infection, and after arthrotomy, we noted that the tibial polyethylene articular component was found to have rotated 180 degrees, with the anterior aspect of the polyethylene facing the posterior aspect of the knee. The polyethylene component was removed. At this time, it was noted that the posterior cruciate ligament had been avulsed off the posterior tibial plateau. The intercondylar notch of the distal femur and posterior aspect of the knee was then debrided. The polyethylene was replaced with the same size component, 5 mm height size 6 rotating-platform polyethylene. It was placed in an appropriate position and was noted to have good range of motion, ligament balance, and anatomic patellar tracking. The patient was, again, discharged without complication.

## 3. Discussion

Rotating-platform knee arthroplasties have been a big innovation in the field of knee replacements in the last three decades. Previous fixed-bearing systems provided a higher level of constraint in order to maintain proper alignment; however, this design was thought to lead to early loosening of TKAs [5]. Designs, such as round-on-flat and flat-on-flat configurations, were aimed at relieving the forces generated onto the prosthesis. The decreased contact area on the polyethylene components led to increased accelerated wear and increased failure of TKAs. Mobile-bearing systems such as rotating-platform TKAs were aimed at correcting these deficiencies. They were originally developed to provide dual-surface articulation that uncouples axial and translational forces of the knee [1]. Uncoupling of these forces was thought to reduce loosening rates of fixed-bearing designs, provide more normal kinematics, and increase survivorship [1]. Although kinematic motion analysis supports the use of mobile-bearing TKAs, clinical studies have failed to report increased efficacy of mobile-bearing TKAs vs. fixed-bearing systems [6].

Dislocation remains a rare, but well-known, complication of mobile-bearing TKAs with incidence at approximately 1% [5, 7]. Risk factors for TKA dislocation including female gender, obesity, and valgus deformity were described by Fisher et al. who documented characteristics of dislocation in 7 out of 1255 cruciate-retaining mobile-bearing TKAs [8]. Additional risk factors include advanced age and previous patellectomy [9]. This suggests that special precaution must be taken in certain patient populations during follow-up of TKA. Turki and Trick and Lee et al. describe two cases of 90-degree dislocation of mobile-bearing TKA seen on radiograph which proceeded to a complete 180-degree rotation following attempted closed reduction [3, 4].

Risk factors of our patient were consistent with morbid obesity (BMI: 51.19), female gender, and advanced age (67), but no preoperative valgus deformity or previous patellectomy. It is possible that the risk factors predisposed our patient to experience spinout. It was also noted, upon revision of the polyethylene, that the patient had avulsed the posterior cruciate ligament off the posterior tibial plateau. This added instability, combined with the force vectors associated with deep flexion while rising to stand could be the reason for the 180-degree spinout.

## 4. Conclusion

Studies of both the kinematics and wear properties of rotating-platform total knee arthroplasty have shown certain benefits compared to fixed-bearing arthroplasties. However, rotating-platform dislocations are a unique complication of mobile-bearing prostheses and occur in a small percentage of cases [7]. We presented a unique case of a spontaneous 180-degree dislocation of the rotating platform. Special attention should be paid to patients with risk factors for severe dislocations such as obesity, age, valgus deformity, and previous surgeries affecting the extensor mechanism. Additionally, it is important to note that closed reduction of partially subluxed polyethylene may lead to a complete 180-degree rotation thus requiring open revision.
